# Molecularly Imprinted Polymer‐Based Smart Prodrug Delivery System for Specific Targeting, Prolonged Retention, and Tumor Microenvironment‐Triggered Release

**DOI:** 10.1002/anie.202012956

**Published:** 2020-12-03

**Authors:** Zikuan Gu, Yueru Dong, Shuxin Xu, Lisheng Wang, Zhen Liu

**Affiliations:** ^1^ State Key Laboratory of Analytical Chemistry for Life Science School of Chemistry and Chemical Engineering Nanjing University 163 Xianlin Avenue Nanjing 210023 China; ^2^ Department of Biochemistry, Microbiology and Immunology Faculty of Medicine University of Ottawa 451 Smyth Road Ottawa Ontario K1H 8M5 Canada

**Keywords:** cancer, drug delivery, molecular imprinting, nanoparticles, polymers

## Abstract

Prodrug and drug delivery systems are two effective strategies for improving the selectivity of chemotherapeutics. Molecularly imprinted polymers (MIPs) have emerged as promising carriers in targeted drug delivery for cancer treatment, but they have not yet been integrated with the prodrug strategy. Reported here is an MIP‐based smart prodrug delivery system for specific targeting, prolonged retention time, and tumor microenvironment‐triggered release. 5′‐Deoxy‐5‐fluorocytidine (DFCR) and sialic acid (SA) were used as a prodrug and a marker for tumor targeting, respectively. Their co‐imprinted nanoparticles were prepared as a smart carrier. Prodrug‐loaded MIP specifically and sustainably accumulated at the tumor site and then gradually released. Unlike conventional prodrug designs, which often require in‐liver bioconversion, this MIP‐based prodrug delivery is liver‐independent but tumor‐dependent. Thus, this study opens new access to the development of smart prodrug delivery nanoplatforms.

## Graphical abstract

A molecularly imprinted polymer (MIP) based smart prodrug delivery nanoplatform was demonstrated with the ability to specifically target tumor sites with prolonged retention time and gradual tumor microenvironmental pH‐triggered release. The MIP‐based prodrug delivery is liver‐independent but tumor‐dependent, which not only greatly enhances tumor specificity but also expands the scope of applicable prodrugs.

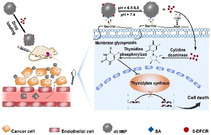

Cancer is the second leading cause of death and a major public health problem around the world.^[1]^ Although chemotherapy has been one mainstream in cancer treatments, it suffers from apparent disadvantages, being nonselective and thereby damaging healthy normal tissues and causing severe side effects. Development of new strategies to tackle this challenge is an unmet medical need. The prodrug strategy in combination with drug delivery systems (DDS) and nanotechnology seems to be a promising new solution. Prodrugs are inactive precursors of active drugs designed to be bioconverted or activated in post administration with improved pharmacokinetic properties of the parent drugs.^[2]^ With increased availability of advanced materials, such as polymers,^[3]^ inorganic carriers,^[4]^ and biomacromolecular scaffolds,^[5]^ DDS has been essential for cancer chemotherapy, capable of specifically delivering chemotherapeutics to tumor sites with enhanced therapeutic efficacy and controlled drug release performance. Nanoscale drug carriers^[6]^ have exhibited the potential to improve treatment efficacy while avoiding toxicity to normal cells, owing to their high selective accumulation in tumors via passive transport through the enhanced permeability and retention (EPR) effect or active transport through conjugating chemotherapeutics‐containing nanocarriers to ligands that can bind with tumor‐specific antigens.

Biomolecules that can recognize specific antigens overexpressed on tumors are key to active drug delivery, including penetrating peptides,^[7]^ aptamers,^[8]^ glycans,^[9]^ monoclonal antibodies,^[10]^ and so on. Molecularly imprinted polymers (MIPs),^[11]^ also referred as plastic antibodies or artificial antibodies, are chemically synthesized through polymerization in the presence of a template, yielding tailormade binding sites complementary to the template molecules in shape, size and functional groups. As compared with biomolecules, MIPs exhibit several significant advantages, including ease of preparation, stability, low cost and simple fabrication into or integration with various nanomaterials. MIPs have shown potential in multiple important application areas, such as separation,^[12]^ sensing,^[13]^ single cell analysis,^[14]^ disease diagnosis,^[15]^ and cancer therapy.^[16]^ Recently, MIPs have emerged as promising alternatives for tumor targeting^[17]^ and targeted drug delivery.^[18]^ However, to the best of our knowledge, MIP‐based smart prodrug delivery systems have not been reported yet.

Herein, we report the rational design of a MIP‐based smart nanocarrier that is capable of specially delivering the active metabolite of a prodrug to tumor sites with improved delivery efficiency and tumor microenvironmental pH‐responsive release. As a proof‐of‐principle, 5‐fluorouracil (FU), which can inhibit DNA synthesis in cells via the inhibition of thymidylate synthase, was used as a parent drug, while 5′‐deoxy‐5‐fluorocytidine (DFCR), which is an inactive precursor of FU, was used as a prodrug in this study. Due to its nonselective cytotoxicity, FU suffers from systemic toxicity, including neutropenia, stomatitis, and diarrhea.^[19]^ To solve these issues, capecitabine has been successfully developed as an orally administered prodrug to treat multiple types of cancer, with improved intratumor drug concentration through three‐step enzymatic conversion to DFCR by carboxylesterase in liver, then to 5′‐deoxy‐5‐fluorouridine (DFUR) by cytidine deaminase in liver/plasma/tumor, and finally to FU by thymidine phosphorylase in tumor.^[20]^ While DFUR had been used as a prodrug of FU,^[21]^ DFCR has not yet been explored and will be investigated in this study as a prodrug. To specifically target tumors, we used sialic acid (SA), which is a monosaccharide overexpressed on cancerous cells,^[22]^ as a tumor marker in this study. MIP nanoparticles (NPs) were prepared as nanocarriers by a state‐of‐the‐art approach called boronate affinity controllable oriented surface imprinting^[23]^ using SA and DFCR as co‐templates. The affinity of the prepared MIP was predominantly governed by the boronic acid ligand used,^[24]^ which provides pH‐dependent affinity towards *cis*‐diol groups. The drug transport and action mechanism are illustrated in Scheme 1. The dual‐templated MIP (dt‐MIP) NPs loaded with DFCR were intravenously injected into experimental nude mice. The DFCR‐loaded dt‐MIP NPs can reach cancer cells via the EPR effect. Meanwhile, due to the affinity towards SA at cancer cell surface, the nanocarriers bind with cancer cells. At the tumor site, which has a slightly acidic microenvironmental pH (6.5–6.8), different binding behaviors occur. The MIP NPs bind with SA on cancer cells because SA can bind with boronic acids at relatively low pH^[25]^ where the loaded prodrug is released gradually from the MIP NPs because acidic pH is unfavorable to the binding between DFCR and boronic acid. The released prodrug is then taken by cancer cells, in which two‐step enzymatic reaction degrades the prodrug into its toxic metabolite and results in cell death. In many regular prodrug designs, liver‐dependent bioconversion is indispensable. As a contrast, the prodrug conversion route in our MIP‐based prodrug delivery is liver‐independent but tumor‐dependent. Such a new approach can not only greatly enhance tumor specificity but also expand the scope of applicable prodrugs.

**Scheme 1:**
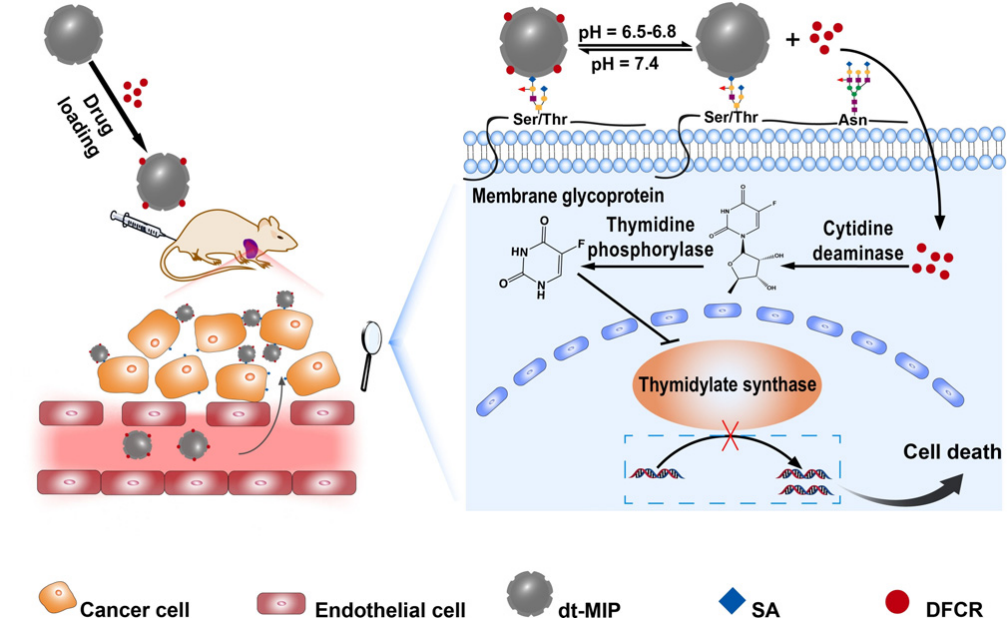
Schematic of the drug transport and action mechanism in the dual‐templated MIP‐based smart prodrug delivery system.

The preparation and drug loading process of dt‐MIP NPs are illustrated in Scheme S2. FITC‐doped NPs were used for in vitro test, while NIR797‐doped ones for in vivo imaging. As shown in Figure S1 (see the Supporting Information), FITC was successfully incorporated in the NPs. 4‐Formylphenylboronic acid (FPBA) was used to modify the NPs. Adenosine and deoxyadenosine were used as test compounds to evaluate if the NPs had been modified with FPBA. Figure S2 confirms successful modification of FPBA on the NPs. Figure S3 and Table S1 confirm the presence of the element N and B in the NPs. The prepared imprinted NPs were well shaped, with a diameter of 60–70 nm (Figure S4). Figure S5 depicts the adsorption isotherms of FPBA‐modified NPs toward both DFCR and SA, indicating that 1 mg FPBA modified NPs could adsorb 1.33 μg DFCR and 1.52 μg SA at the same time. These data suggest that the FPBA‐NPs could bind the two templates of adequate amount at the same time.

We first optimized the imprinting time for DFCR and evaluated the imprinting effect in terms of imprinting factor (IF). IF is determined by the ratio of the amount of target compound captured by the MIP over by non‐imprinted polymer (NIP) prepared using a similar procedure without template immobilization. Figure 1 A shows that the imprinting for 15 min gave the best IF value (6.8). Since previous studies^[26]^ have indicated that the best imprinting time for SA was 20 min using the same imprinting approach and the specificity toward SA is more critical and determines the tumor targeting specificity, 20 min was selected to prepare dt‐MIP to ensure the tumor targeting specificity with slight sacrifice in the affinity towards DFCR. For the imprinting time of 20 min, the IF value (4.8) was the second best, which is well accepted. Such a high IF value means that the MIP had higher affinity as compared with NIP. The prepared dt‐MIP exhibited good selectivity (Figure 1 B).

**Figure 1:**
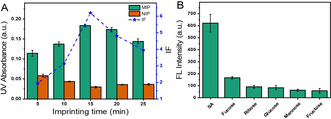
A) Effect of imprinting time on the binding capability of DFCR‐imprinted and non‐imprinted NPs towards DFCR; B) Selectivity of dt‐MIP prepared with imprinting time of 20 min towards different monosaccharides.

To measure the drug loading capacity of dt‐MIP and binding affinity, we investigated the adsorption isotherms. As shown in Figure S6, as increasing the concentration of DFCR or SA, the amount of test compound bound by dt‐MIP gradually increased and reached saturation at a certain range of concentration; when the concentration further increased, the amount bound also further increased. We explain these results by the co‐existence of two types of imprinted cavities; one for DFCR and the other for SA. At low concentration to saturation, the test compound bound with its own imprinted cavities, due to well‐matched shape and higher affinity; when the concentration was higher than the saturation concentration, the remained cavities, which were produced with the other template, could also adsorb some more amount of the test compound, due to relatively weak affinity of the boronate ligand existed in the imprinted cavities. The drug loading capacity of dt‐MIP was found to be 1.18 μg mg^−1^ for DFCR. Based on the isotherms, the association constants (*K*_d_) of dt‐MIP towards DFCR and SA were estimated to be 1.14×10^−3^ M and 1.43×10^−4^ M, respectively.

Drug releasing kinetics at different pH was then tested. To simulate the microenvironment in tumor tissue and normal tissue, we used phosphate buffers of two pH values: pH 7.4 for simulating the environmental pH in blood circulation and normal tissue while pH 6.8 for simulating the microenvironmental pH in tumor tissue. At pH 7.4, DFCR will not be released from the cavity due to the strong and undisturbed covalent bond between the boronic acid and DFCR. At pH 6.8, however, the covalent bond between the boronic acid and DFCR is slightly disrupted and DFCR can be gradually released. As shown in Figure 2 A, at pH 6.8, DFCR release ratio increased gradually over time, reaching 55 % at 120 h; in contrast, DFCR release ratio was tightly constrained at pH 7.4.

**Figure 2:**
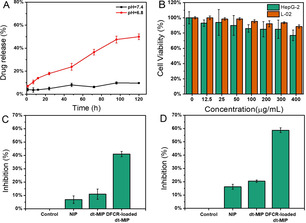
A) Release profiles of DFCR in DFCR‐loaded dt‐MIP at pH 7.4 and pH 6.8; B) Cell viability of liver cancer HepG‐2 cells and normal control L‐02 cells treated with different concentrations of dt‐MIP; C) and D) Inhibition of HepG‐2 cell growth by different materials (200 μg mL^−1^) at 24 h (C) and 48 h (D).

The cytotoxicity of the MIP to normal cells was investigated by the 3‐(4,5‐dimethylthiazol‐2‐yl‐)‐2,5‐diphenylterazolium bromide (MTT) assay. Because cytidine deaminase and thymidine phosphorylase are highly expressed in hepatic cancer and breast cancer,^[27]^ we chose HepG‐2 and MCF‐7 cells for the inhibition activity test. Equivalent numbers of cells were incubated with NIP, dt‐MIP, and DFCR‐loaded dt‐MIP, respectively. As shown in Figure 2 B and Figure S7A, when the concentration was lower than 400 μg mL^−1^, the dt‐MIP was nearly nontoxic to both normal cells and cancer cells. As shown in Figure 2 C and 2 D, the DFCR‐loaded dt‐MIP inhibited the growth of HepG‐2 cells to 41 % and 58 % after incubation for 24 h and 48 h, respectively. As a comparison, the NIP and dt‐MIP groups exhibited much lower inhibition capability. Similar inhibition results were observed for MCF‐7 cells (Figure S7B). We then compared the activities of DFCR and DFCR‐loaded dt‐MIP in HepG‐2. As shown in Figure S7C and S7D, the IC_50_ values were determined to be 0.51 and 73 μM for DFCR‐loaded dt‐MIP and DFCR, respectively. The enhanced drug cytotoxicity can probably be attributed to dt‐MIP‐mediated endocytosis.

Although the selectivity of SA‐imprinted MIP prepared by the same imprinting approach towards cancer cells has been verified in the previous studies,^[26]^ the selectivity of dt‐MIP to cancer cells versus normal cells has not yet been tested and compared. As shown in Figure S8, after being stained with FITC‐doped dt‐MIP for 30 min, MCF‐7 and HepG‐2 cancer cell lines showed strong fluorescence, while MCF‐10A and L‐02 normal cell lines showed limited fluorescence. Meanwhile, all the cell lines showed almost no fluorescence after being stained with FITC‐doped NIP. The flow cytometry quantitative analysis shown in Figure S8 verified the selectivity of the dt‐MIP towards SA overexpressed cells (HepG‐2 and MCF‐7) over normal cells (L‐02 and MCF‐10A). These results indicate that the MIP specifically targeted cancer cells through binding with SA overexpressed on cancer cells.

We further investigated the in vivo targeting capability and antitumor activity of the MIP in nude mice xenografted with HepG‐2 cells. After intravenous injection with NIR797‐doped dt‐MIP, SA‐MIP, DFCR‐MIP, NIP as well as PBS for different times, fluorescence imaging was performed to display biodistribution of the injected substances (Figures 3 and S9). For mice injected with PBS, no fluorescence signal was observed all the time, which is reasonable since no fluorescent substance was injected. For mice injected with dt‐MIP and SA‐MIP, the injected materials started to appear at the tumor site at 12 h and then accumulated up to 10 days. While accumulating quickly at the liver area within 2 h to 2 days, dt‐MIP and SA‐MIP gradually declined. Particularly, at day 9 and later on, a significant amount of injected dt‐MIP and SA‐MIP still remained at the tumor site but completely disappeared at the liver site. Such tumor targeting capability and prolonged retention time are assigned to the binding of the MIPs towards SA overexpressed on the tumor. For the mice injected with NIP, the material was mainly located at the liver and lasted for 8 days; only a limited amount was accumulated at the tumor site and lasted for a short period (from 3 days to 5 days). The short‐term and limited residence of NIP at the tumor site may associate with the EPR effect.^[28]^ For the mice injected with DFCR‐MIP, the injected DFCR‐MIP mainly resided at the liver and started to slowly accumulate at the tumor site after 3 days; the resided time at the tumor site was as long as dt‐MIP and SA‐MIP did. The prolonged residence of DFCR‐imprinted MIP at the tumor site compared with NIP was probably due to the boronate affinity of DFCR‐imprinted cavities towards SA on the tumor. To study quantitative biodistribution in the main organs, we sacrificed the mice treated with these materials for 7 days and collected the main organs (heart, liver, spleen, lung, kidney) and the tumor for imaging. Although all the materials predominantly accumulated in the liver, only dt‐MIP and SA‐MIP accumulated in the tumor site for a great extent, while the biodistribution of all kinds of the materials in other organs was limited (Figure S10). The high distribution of dt‐MIP and SA‐MIP at the tumor site can be attributed to the affinity of SA‐imprinted cavities towards SA expressed on the tumor.

**Figure 3:**
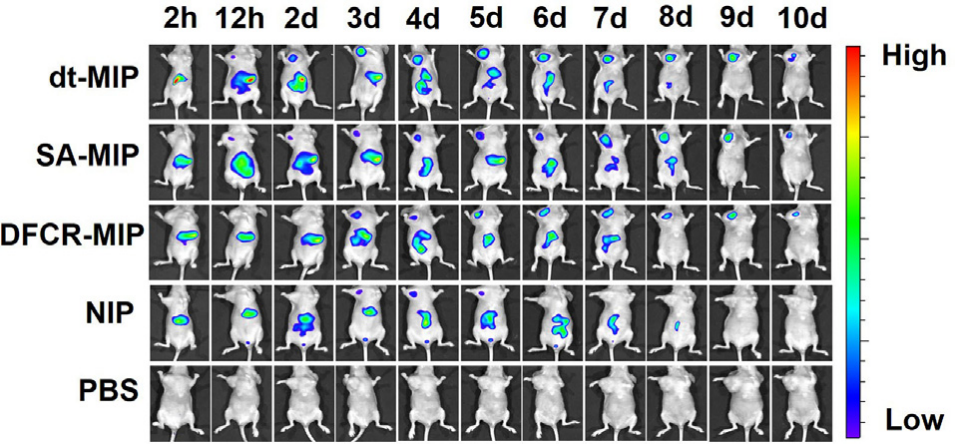
In vivo fluorescence imaging of HepG‐2 tumor (left upper chest) and liver site (upper abdomen) after intravenous injection of NIR797‐doped dt‐MIP, SA‐MIP, DFCR‐MIP, NIP and PBS for different times.

Antitumor activity of the MIP in nude mice xenografted with HepG‐2 cells was then investigated. Figure 4 A shows the change in the tumor volume of the mice after different treatments. The groups treated with PBS, DFCR and dt‐MIP showed fastest tumor growth with similar speed. This suggests that when DFCR was not delivered appropriately, it had almost no antitumor activity. However, the tumor growth speed of the groups treated with DFCR‐loaded DFCR‐MIP and DFCR‐loaded dt‐MIP was much slower than that of PBS treated group, suggesting that the DFCR‐loaded materials inhibited the tumor growth. Among all the treatments, DFCR‐loaded dt‐MIP was the most effective in the delay of tumor growth. This may be attributed to the fact that the dt‐MIP could specifically deliver the drug to the tumor site with the prolonged tumor retention time.

**Figure 4:**
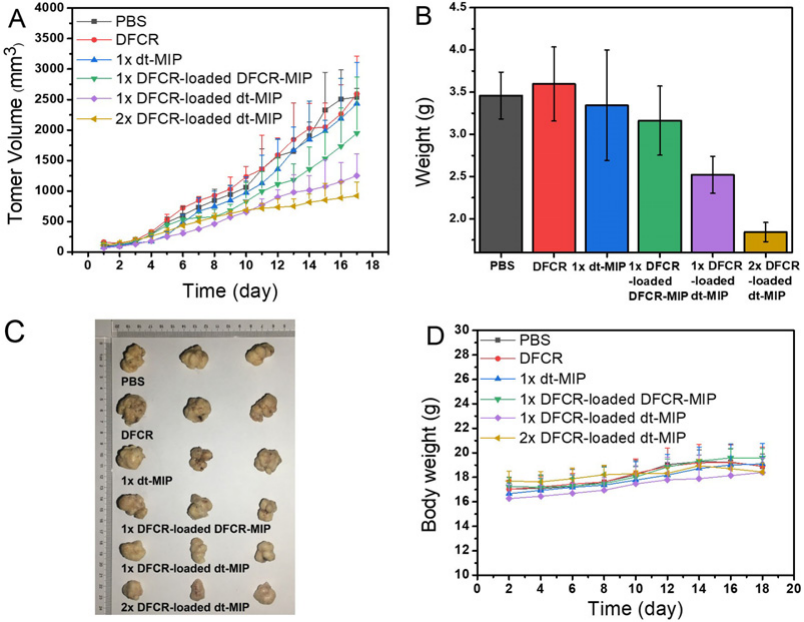
A) Mean tumor volume. B) Mean tumor weights after excision at 18 days. C) Representative photographs of mice in different groups after treatment for 18 days. D) Body weight of the mice in different groups at different time intervals.

We further asked whether DFCR‐loaded dt‐MIP exhibited a dose‐response pattern, which is one of important characteristics in drug development. A double amount of DFCR‐loaded dt‐MIP was injected intravenously. As expected, tumor growth was further delayed (Figure 4 A). The tumor weight and tumor size at day 18 after different treatments were showed in Figure 4 B and Figure 4 C. Of note, there was no significant change in mouse body weight amongst different groups (Figure 4 D), and no obvious side effects observed during the whole experiment.

Finally, histopathologic assessment of mouse liver tissues after treatments was performed. As shown in Figure S11, the Hematoxylin and Eosin (H&E) staining of the liver tissues did not show significant pathological changes in DFCR‐loaded dt‐MIP group compared with PBS group. This suggests that the DFCR‐loaded dt‐MIP does not have hepatotoxicity and higher doses could be administered to further improve in vivo treatment efficacy. It is noteworthy that the DFCR group also did not exhibit significant pathological change, which implies that DFCR did not result in apparent liver toxicity under the experimental conditions in this study. This is in agreement with the clinical report that apparent acute liver injury due to capecitabine therapy has rarely observed.^[29]^ As such, our new approach may hold great potential for cancer treatment.

In summary, we have demonstrated boronate affinity‐based dual‐template imprinted MIP NPs as a promising nanoplatform for smart delivery of DFCR, an inactive precursor of FU that has not been explored as a prodrug before. The molecularly imprinted nanocarriers exhibited not only the ability to specifically target tumor site with prolonged retention time and tumor microenvironmental pH‐triggered gradual release, but also new potential. Since this MIP‐based smart prodrug delivery system is liver‐independent but tumor‐dependent, it is not only more specific to target cancers but also more flexible in prodrug selection. Therefore, this study opened a promising new avenue for the development of smart prodrug delivery system. Along with the imprinting approach used in this study, many imprinting approaches, for example, epitope imprinting^[30]^ and Pickering emulsion‐based imprinting,^[31]^ have enabled facile production of MIPs to target tumor‐specific proteins. Thus, the molecular imprinting technology holds great potential in smart prodrug delivery for cancer therapy.

## Conflict of interest

The authors declare no conflict of interest.

## Supporting information

As a service to our authors and readers, this journal provides supporting information supplied by the authors. Such materials are peer reviewed and may be re‐organized for online delivery, but are not copy‐edited or typeset. Technical support issues arising from supporting information (other than missing files) should be addressed to the authors.

Supplementary
