# Supplementary material for: Molecularly Imprinted Polymer‐Based Smart Prodrug Delivery System for Specific Targeting, Prolonged Retention, and Tumor Microenvironment‐Triggered Release
Source: Angew Chem Int Ed Engl. 2020 Dec 3;60(5):2663–7. doi: 10.1002/anie.202012956 (PMC7898932; doi:10.1002/anie.202012956)
Supplement: Supplementary file 1 — Supplementary [file ANIE-60-2663-s001.pdf]

Angewandte Chemie
Eine Zeitschrift der Gesellschaft Deutscher Chemiker

Supporting Information

Molecularly Imprinted Polymer-Based Smart Prodrug Delivery System for Specific Targeting, Prolonged Retention, and Tumor Microenvironment-Triggered Release

*Zikuan Gu[+], Yueru Dong[+], Shuxin Xu, Lisheng Wang, and Zhen Liu**

anie_202012956_sm_miscellaneous_information.pdf

Experimental Section

Reagents and materials.

3-Aminopropyltriethoxysilane (APTES, 98%), fluorescein isothiocyanate isomer I (FITC), and NIR797 isothiocyanate were purchased from Sigma Aldrich (St. Louis, MO, USA). 5’-Deoxy-5-fluorocytidine (DFCR), N-Acetyineuraminic acid (SA), adenosine and deoxyadenosine were from Aladdin (Shanghai, China). Sodium cyanoborohydride ($NaBCNH_3$), tetraethyl orthosilicate (TEOS) and 4-formylphenylboronic acid (FPBA), D-Fructose, D-Mannose, D-Glucose were purchased from J&K scientific (Shanghai, China). Female SPF mice (4 weeks, 20-25 g), mammary cancer cell MCF-7, normal mammary epithelial cell MCF-10A, hepatoma carcinoma cell HepG-2, normal hepatocyte cell L-02, phosphate-buffered saline solutions for cell culture (1x PBS), parenzyme cell digestion solution (containing 0.25% trypase and 0.02% EDTA), Dulbecco Modified Eagle Medium (DMEM, containing 4.5 mg/mL glucose, 80 U/mL penicillin and 0.08 mg/mL streptomycin), Roswell Park Memorial institute 1640 medium (RPMI-1640, containing 2.0 mg/mL D-glucose, 0.3 mg/mL glutamine, 2.0 mg/mL $NaHCO_3$, 80 U/mL penicillin, and 0.08 mg/mL streptomycin), dimethyl sulfoxide (DMSO, 99%), 3-(4.5-Dimethylthiazol-2-yl)-2,5-diphenyltertrazolium bromide (MTT), 96-well plates were purchased from Keygen Biotech (Nanjing, China). Fetal bovine serum (FBS) and calf serum were purchased from Gibco (Life Technologies, Australia). The mice were acclimatized for 7 d after arrival. All the mice were housed in plastic cages with free access to food and water. All the animal experiments were performed in accordance with the ethical guidelines approved by the Animal Care Committee of the Affiliated Drum Tower Hospital of Nanjing University Medical School.

Instruments.

Transmission electron microscopic (TEM) characterization was performed on a JEOL JEM-2100 TEM instrument (Tokyo, Japan). UV-vis absorption was recorded on a NanoDrop spectrometer from ThermoFisher Scientific (Waltham, MA, USA). Fluorescence characterization and evaluation of boronic acid functionalization and the imprinting procedure were performed on a SynergyMX from BioTek (Winooski, VT, USA). Cell imaging was performed on a Leica TCS SP5 confocal laser scanning microscope (Leica Microsystems CMS GmbH, Mannheim, Germany). In vivo imaging experiment was performed on a PerkinElmer Caliper IVIS Lumina XR III in vivo imaging system (Waltham, Massachusetts, USA). Flow cytometry was performed on Flow cytometer (Beckman Coulter FC).

Synthesis of FITC-doped SiO_2 NPs.

The preparation of FITC-doped SiO_2 NPs (FITC-doped NPs) included two steps: 1) the synthesis of FITC-derivatized APTES, and 2) the polycondensation of FITC-derivatized APTES with TEOS. The amino group of APTES can react with the isothiocyanate of FITC to yield a thiourea bridge. A volume of 50 μL APTES and 10 mg FITC were dissolved in 10 mL of ethanol. After reaction in darkness with vigorous stirring for 10 h,

FITC-derivatized APTES was formed in the solution. 200 mL of ethanol was mixed with 12.125 mL of deionized water and 9 mL of ammonium hydroxide. The solution was heated to 55°C with vigorous stirring and then the precursor solution was added rapidly.The precursor was composed of 6.25 mL of FITC-derivatized APTES solution, 1.5 mL of TEOS and 20 mL of ethanol. After reaction for about 50 min, FITC-doped NPs were formed and collected by centrifugation at 11400 rpm for 15 min. Finally, the collected nanoparticles were washed with ethanol twice, dried and then weighted. The obtained nanoparticles were dispersed in ethanol with final concentration at 10 mg/mL for later use.

Preparation of FITC-doped dual-templated MIP (dt-MIP) NPs.

FITC-doped dual-templated MIP NPs were prepared according to a method adapted from the boronate affinity controllable oriented surface imprinting approach reported previously.[1] The synthesis procedure included four steps: 1) the preparation of FPBA-functionalized FITC-doped NPs, 2) the immobilization of SA and DFCR onto FPBA-functionalized FITC-doped NPs, 3) controllable oriented imprinting, and 4) template removal. Firstly, to prepare FPBA-functionalized FITC-doped NPs, FITC-doped NPs were dispersed in 1 mL of ethanol solution, followed by the addition of 5 mg FPBA and 1 mg sodium cyanoborohydride. After reaction at room temperature for 12 h, the FPBA-functionalized FITC-doped NPs were collected by centrifugation, and then were washed with ethanol and water for three times, respectively. To immobilize SA and DFCR, 10 mg of FPBA-functionalized nanoparticles were re-dispersed into 10 mL of phosphate buffer (0.01 M, pH = 7.4) and the nanoparticle concentration is 1 mg/mL. Then 10 mg of SA and 10 mg of DFCR were added into the dispersion. After incubation for 2 h, template-binding FITC-doped NPs were collected via centrifugation and then washed with 0.01 M phosphate buffer (pH 7.4) for three times. The template-binding FITC-doped NPs were re-dispersed withou extra drying into 10 mL of ethanol and then added with 0.175 mL of ammonium hydroxide and 2.5 mL of precusor consisted of 56 μL TEOS and 25 mL ethanol. After reaction for an appropriate duration, the dispersion was centrifuged and the precipitates were collected. The collected NPs were washed with 5 mL of 0.1 M HAc for 2 h to remove the temple. Finally, the imprinted NPs were dispersed in 0.01 M phosphate buffer (pH 7.4). To prepare FITC-doped non-imprinted NPs (NIP) for comparison, the procedure was the same except that no template was immobilized onto FPBA-functionalized FITC-doped NPs.

Preparation of DFCR-imprinted NPs (DFCR-MIP).

The process of preparation of DFCR-imprinted NPs was the same as the preparation of FITC-doped dual-templated MIP NPs except that only DFCR was used as the template.

Preparation of SA-imprinted NPs (SA-MIP).

The process of preparation of SA-imprinted NPs was the same as the preparation of FITC-doped dual-templated MIP NPs except that only SA was used as the template.

Boronate affinity sandwich assay.

In order to evaluate boronic acid-functionalization of FITC-doped NPs and relevant properties of SA-and DFCR-imprinted FITC-doped NPs in terms of imprinting factor (IF) and the selectivity, the boronate affinity sandwich assay[2] was performed. Briefly, a boronic acid-modified 96-well microplate was used as a substrate, and the template molecules such as SA was used as a bridge compound. The bridge molecule can bind the substrate and the imprinted NPs. After excessive nanoparticles were removed through washing with phosphate buffer (0.01 M, pH 7.4), the fluorescence signal was detected on the SynergyMX microreader. The boronic acid-modified 96-well microplate was prepared according to a previous method.[2-4] Firstly the wells were filled with a 3:1 (v/v) mixture of H_2SO_4 (98%) and HNO_3 (63%) (250 µL/well) and kept at room temperature for 12 h. After being washed with deionized water to achieve a neutral pH, the wells were air-dried. After that, the wells were filled with 5% aqueous APTES solution (pH 6.9, 250 µL/well), slightly shaken at room temperature for 2 h, and then dried by air. The wells were then filled with methanol solution containing 5 mg/mL 4-formylphenylboronic acid and 5 mg/mL sodium cyanoborohydride dissolved in anhydrous methanol (250 µL/well). The microplate was sealed and slightly shaken at room temperature for 12 h, and then the wells were washed with ethanol for 5-10 times. The obtained microplate was dried by air and then kept at 4ºC for later experiments.

Characterization of boronic acid-modified 96-well microplate.

To characterize the modification of boronic acid onto the 96-well microplate, the glycoprotein HRP was used as a test compound. Three random boronic acid-modified wells and three unmodified wells were added with 250 µL of 0.5 mg/mL HRP dissolved in 0.01 M phosphate buffer (pH 7.4). The three unmodified wells filled with equal volume phosphate buffer with HRP were used as controls. After incubation for 30 min, the wells were washed with 0.01 M phosphate buffer (pH 7.4) three times. The UV absorbance at 260 nm of each well was read on the SynergyMX microreader.

Evaluation of boronic acid-functionalization of FITC-doped NPs.

The boronic acid functionalization of FITC-doped NPs was evaluated according to the boronate affinity of the prepared material using the cis-diol group containing compound adenosine as a test compound and non-cis-diol containing compound deoxyadenosine as a control. FPBA-fuctionalized NPs were dispersed in 0.01 M phosphate buffer (pH 7.4) to make the final concentration of 1 mg/mL. A volume of 1 mL of 1 mg/mL boronic acid-functionalization NPs was incubated with 1 mg adenosine or deoxyadenosine dissolved in 0.01 M phosphate buffer (pH 7.4) for 1 h. After that, the NPs were collected via centrifugation and washed with 0.01 M phosphate buffer (pH 7.4) for three times. The collected NPs were further eluted with 40 µL of 0.1 M HAc for 30 min. Finally, the supernatant was collected via centrifugation and tested by UV absorbance at 260 nm.

Calibration curves of DFCR and SA.

A series of standard mixed solution of SA and DFCR (0.1, 0.2, 0.3, 0.4, 0.5, 0.6, 0.7, 0.8 mg/mL for SA as well as DFCR) were prepared with 0.01 M phosphate buffer (pH 7.4) and the absorbance at 284 nm, 200 nm were measured. At 284 nm, there is no UV absorbance for SA, and the calibration cruve of the mixed solutions at 284 nm can be used for the quantification of DFCR. The absorbance at 200 nm of the mixed solution is the cumulation of SA and DFCR. A series of standard solution of DFCR solutions (0.1, 0.2, 0.3, 0.4, 0.5, 0.6, 0.7, 0.8 mg/mL for DFCR) were prepared with 0.01 M phosphate buffer (pH 7.4) and the absorbance at 200 nm was measured. The difference value of absorbance at 200 nm between the mixed solutions and DFCR at 200 nm were used for fitting the calibrartion cruve of SA.

Adsorption isotherm of boronic acid-functionalization of FITC-doped NPs to the SA and DFCR.

A series of standard mixed solution of SA and DFCR (0.2, 0.3, 0.4, 0.5, 0.6, 0.7, 0.8 mg/mL SA as well as DFCR mixed solutions) were prepared with 0.01 M phosphate buffer (pH 7.4). The absorbance at 284 nm and 200 nm were measured. 5 mg of boronic acid-functionalized NPs were incubated with 100 μL of these mixed solutions, respectively. After incubation for 30 min, all the dispersions were centrifuged and the absorbance of the supernatants at 284 nm, 200 nm were measured. The amounts of DFCR captured by the NPs were calculated by the difference between the absorbance for initial mixed solutions and the corresponding supernatants at 284 nm. The SA amounts captured by the NPs were calculated by the difference between the difference signal for initial mixed solutions and the corresponding supernatants at 200 nm and the signal of DFCR.

Optimization of the imprinting time of DFCR.

DFCR imprinted NPs were prepared according to the boronate affinity oriented surface imprinting approach reported previously[1] with modifications. 4 mg of DFCR was added into 4 mL of FPBA-functionalized NPs dispersion (1 mg/mL) in 0.01 M phosphate buffer (pH 7.4). After incubation for 2 h, DFCR-bound NPs were collected via centrifugation and washed with 0.01 M phosphate buffer (pH 7.4) for three times. The template-binding FPBA-functionalized NPs were re-dispersed into 4 mL of ethanol and then added with 0.07 mL of ammonium hydroxide and then added with 1 mL of monemer solution with 22.4 μL of TEOS in 10 mL of ethanol. After reaction for 5, 10, 15, 20 or 25 min, the dispersion was centrifuged and the precipitates were collected. The collected NPs were washed with 1 mL of 0.1 M HAc for 2 h to remove the temple. At last, the imprinted NPs were collected by centrifugation and dispersed in 4 mL of 0.01 M phosphate buffer (pH 7.4). To prepare non-imprinted NPs for comparison, the processing procedure was the same except that no template was immobilized onto FPBA-functionalized NPs.

The imprinting time was optimized in terms of the imprinting factor (IF) obtained with different imprinting times. 1 mL of dispersion containing 1 mg of imprinted or non-imprinted NPs were prepared to test the IF value. 1 mg of DFCR was added in the dispersions. After incubation for 1 h, the NPs were collected via centrifugation

and then eluted with 40 μL of 0.1 M HAc with shaking for 40 min. The IF value was determined by testing the UV absorbance at 284 nm of the supernatants.

SA selectivity test of the dt-MIP NPs.

A boronic acid-functionalized 96-well microplate was used for the experiments. The wells were added with equivalent volumes of six different solutions and incubated for 1 h: (A1-C1) and (A2-C2) 0.01 M phosphate buffer (pH 7.4) containing 1 mg/mL SA; (A3-C3) and (A4-C4) 0.01 M phosphate buffer (pH 7.4) containing 1 mg/mL D-Fru; (A5-C5) and (A6-C6) 0.01 M phosphate buffer (pH 7.4) containing 1 mg/mL D-Man; (A7-C7) and (A8-C8) 0.01M phosphate buffer (pH 7.4) containing 1 mg/mL L-Fuc; (A9-C9) and (A10-C10) 0.01M phosphate buffer containing 1 mg/mL D-Glu; (A11-C11) and (A12-C12) 0.01 M phosphate buffer containing 1 mg/mL D-Rib. After incubation for 1 h, the wells were washed with 0.01 M phosphate buffer (pH 7.4) three times and then (A1-C1); (A3-C3), (A5-C5), (A7-C7), (A9-C9), and (A11-C11) were added with equivalent volume of 0.01 M phosphate buffer (pH 7.4) containing 1 mg/mL FITC-doped dt-MIP NPs and (A2-C2); (A4-C4), (A6-C6), (A8-C8), and (A10-C10) were added with equivalent volume of 0.01 M phosphate buffer (pH 7.4) containing 1 mg/mL FITC-doped non-imprinted NPs and incubated for another 40 min. Thereafter, the wells were washed with 0.01 M phosphate buffer (pH 7.4) three times. Finally, the fluorescence of the wells were read on the SynergyMX and the fluorescence intensity was control-subtracted and averaged over each column.

Adsorption isotherm of dt-MIP NPs to DFCR.

A series of standard solutions of DFCR (0.2, 0.3, 0.4, 0.5, 0.6, 0.7, 0.8, 0.9, 1.0 mg/mL) were prepared with 0.01 M phosphate buffer (pH 7.4) and the absorbance at 284 nm was measured. 5 mg of dt-MIP NPs were incubated with 100 μL of these solutions, respectively. After incubation for 30 min, all the solutions were centrifuged and the absorbance of supernatants at 284 nm were measured. The amounts of DFCR captured by the dt-MIP NPs were calculated by the difference value of absorbance at 284 nm between the initial solutions and the corresponding supernatants.

Adsorption isotherm and binding constant measurement of dt-MIP NPs to SA.

A series of standard solutions of SA (0.025, 0.05, 0.1, 0.2, 0.3, 0.4, 0.5, 0.6 mg/mL) were prepared with 0.01 M phosphate buffer (pH 7.4) and the absorbance at 200 nm were measured. 5 mg of dt-MIP NPs were incubated with 100 μL these solutions, respectively. After incubation for 30 min, all the dispersions were centrifuged and the absorbance at 200 nm were measured. The amounts of SA captured by the NPs were calculated by the difference value of absorbance at 200 nm between the initial solutions and the corresponding supernatants. The Hill equation as shown below was used for fitting the dissociation constant (K_d).

$$Q_e = Q_{max} x^n / (x^n + K_d^n) \qquad (1)$$

where Q_e is the amount of the target compound bound by the dt-MIP at equilibrium, Q_{max} is the saturated adsorption capacity, *x* is the concentration of free target compound, and *n* is and the Hill coefficient.

In vitro cytotoxicity of FITC-doped dual-templated MIP NPs.

MCF-7 and MCF-10A cells were cultured in RPMI-1640 medium with 10% fetal bovine serum (37°C, 5% CO_2), while HepG-2 and L02 cells were cultured in DMEM medium with 10% fetal bovine serum before use. Cell viability was determined by the MTT assay. Briefly, MCF-10A, L02, MCF-7, HepG-2 were harvested and placed in 96-well plates at concentration of 5,000 cells/well. The cells were incubated with different concentrations of FITC-doped dual-templated MIP NPs at 37 °C for 24 h. The cells without NPs were used as control and others were used as test group. And wells without cells were used as background group. Then 50 μL of MTT indicator dye (1 mg/mL in 0.01 M phosphate buffer, pH 7.4) was added. After incubating for another 4 h at 37°C in the dark, the supernatant was discarded, and 150 μL of DMSO was added to each well. After shaking for ten minutes on the shaking table, the optical density of the solution was monitored on the Varioskan Flash.

Absorbance was measured at a wavelength of 550 nm. The cell viability was expressed as a percentage of the absorbance of test cells (added with NPs) over that of control experiment (without any NPs) (both were deducted by the background absorbance), which can be calculated by the following equation:

$$\text{Cell viability (\%)} = \frac{\text{Abs (test)} - \text{Abs (background)}}{\text{Abs (control)} - \text{Abs (background)}} \times 100\% \quad (2)$$

Proliferation inhibition test in vitro and IC_{50} calculation.

Exponentially growing HepG-2 were harvested and placed in 96-well plates at concentration of 5,000 cells/well. After incubation at 37°C for 24 h, the HepG-2 cells were respectively treated with DFCR and DFCR-loaded dt-MIP dispersed in cell culture medium with different concentrations (the concentration of DFCR is from 0 μg/mL-200 μg/ mL, and the concentration of DFCR-loaded dt-MIP is from 0 μg/mL-2 mg/ mL) for 24 h. The cells without NPs were used as control. The wells with equal volume of cell culture medium were used as background. Then 50 μL of MTT (1 mg/mL) was added to each well and the plates were incubated at 37°C for another 4 h in dark. Subsequently, the supernatant was discarded and 150 μL of DMSO was added to each well. After shaking for 10 min, the absorption of the solution was monitored on a Varioskan Flash.

Absorbance at 550 nm was measured. Inhibition of cell growth was calculated according to the following equation:

$$\text{Inhibition (\%)} = \frac{(\text{OD}_{\text{control}} - \text{OD}_{\text{treatment}})}{(\text{OD}_{\text{control}} - \text{OD}_{\text{background}})} \times 100\% \quad (3)$$

Data are reported as the mean of three independent experiments.

The IC_{50} value of tested drug was obtained by fitting the data according to the following equation:

$$Y = Min + \frac{Max-Min}{1+ \left(\frac{X}{IC_{50}}\right)^{h}} \quad (4)$$

where X is the drug concentration, Y is the cell vialibity%, and h is the Hill coefficient. *Min* stands for the minimum *Y* value, while *Max* stands for the maximal *Y* value.

Proliferation inhibition test in vitro for different time.

Exponentially growing MCF-7, HepG-2 were harvested and placed in 96-well plates at concentration of 5,000 cells/well. After incubation at 37°C for 24 h, the HepG-2 and MCF-7 cells were respectively treated with DFCR-loaded dt-MIP dispersed in cell culture medium at 200 μg/mL for 24h and 48h. The group without NPs was used as control. The wells with equal volume of cell culture medium were used as background. Then 50 μL of MTT (1 mg/mL) was added to each well and the plates were incubated at 37°C for another 4 h in dark. Subsequently, the supernatant was discarded and 150 μL of DMSO was added to each well. After shaking for 10 min, the absorption of the solution was monitored on a Varioskan Flash. Absorbance at 550 nm was measured and inhibition of cell growth was calculated according to Eq (3). Data were reported as the mean of three independent experiments.

Cell culture and imaging.

The cell culture medium was removed and the cells remained on the cell culture dishes were washed with 1xPBS twice. Then cells were respectively incubated with 200 μL of FITC-doped dual-templated MIP NPs, FITC-doped non-imprinted NPs dissolved in 1XPBS (200 μg/mL each) for 30 min. Subsequently, the PBS buffer and free nanoparticles were removed and the remaining cells were rinsed with 1XPBS for three times. The obtained cells were imaged under the confocal laser-scanning microscope.

Flow cytometry.

For flow cytometry assay, the MCF-7, MCF-10A, HepG-2, L-02 cells were respectively stained with 2 mL of FITC-doped dual-templated MIP NPs and FITC-doped non-imprinted NPs dissolved in 0.01 M phosphate buffer (pH 7.4) (200 μg/mL for each) for 30 min. Then the cells were digested with parenzyme cell digestion solution (containing 0.25% tryptase and 0.02% EDTA) for 2-3 min. The obtained cells were centrifuged at 1000 rpm for 3 min. After removing the supernatant, the cells were disappeared in 1.5 mL of 0.01 M phosphate buffer (pH 7.4). Before injected the cells into cytoanalyzer, the cells were filtrated with 200 mesh sieves, and the count of cells was set to 10,000.

Preparation of NIR797-doped dual-templated NPs.

NIR797-doped dual-templated MIP NPs were prepared as the same as the preparation of FITC-doped NPs described above except that the NIR797 dye was reacted with APTES.

In vivo biodistribution in the mice.

All the animal experiments were approved by the Animal Care and Use Committee of Nanjing University. The accreditation number of the investigator was 220200448. A total of 25 female HepG-2 tumor-bearing mice with volume of approximately 150 mm^3 were divided into 5 groups and injection with different materials: 1) NIR797-doped dt-MIP; 2) NIR797-doped SA-MIP; 3) NIR797-doped DFCR-MIP; 4) NIR797-doped NIP; 5) 1XPBS (control). The injection does was about 1.5 mg/every mice. The distribution of NPs in mice was imaged using the PerkinElmer in vivo imaging system. The mice were anesthetized with isoflurane and images were taken at different times. After 7 days, three mice of every group were dissected, the main organs (hearts, liver, spleen, lung, kidney) and tumor were collected for fluorescence imaging and quantative biodistribution. The remaining mice of every group were imaged until 10th day.

In vivo antitumor activity.

A total of 30 female HepG-2 tumor-bearing mice with volume of approximately 50 mm^3 were randomly divided into 6 groups and injected with different materials once every four days: 1) PBS; 2) 1 mg/mL DFCR; 3) 10 mg/mL dt-MIP; 4) 10 mg/mL DFCR-loaded DFCR-MIP; 5) 10 mg/mL DFCR-loaded dt-MIP; 6) 10 mg/mL DFCR-loaded dt-MIP. The injection dose of the group 1, 2, 3, 4, 5 was about 150 μL/every mouse, and the injection does of the group 6 was about 300 μL/every mouse. A caliper was applied to measure the volume of the tumor each day. The tumor volume V was calculated based on the formula, $V = Dd^2/2$, where D and d refer to the length and width of the tumor. The body weights of mice were measured by an electronic balance every two days. On the 18th day, the mice were dissected and the tumor were collected for photopraphing and weighting, and after that H&E staining of liver were also performed.

References

[1] R. R. Xing, S. S. Wang, Z. J. Bie, Z. Liu, *Nat. Protoc*. **2017**, *12*, 964-987.
[2] J. Ye, Y. Chen, Z. Liu, *Angew. Chem. Int. Ed.* **2014**, *53*, 10386-10389.
[3] S. S. Wang, D. Y. Yin, W. J. Wang, X. J. Shen, J. J. Zhu, H. Y. Chen, Z. Liu, *Sci. Reports* **2016**, *6*, 22757.
[4] S. S. Wang, Y. R. Wen, Y. J. Wang, Y. Y. Ma, Z. Liu, *Anal. Chem.* **2017**, *89*, 5646−5652.

Supplementary Data

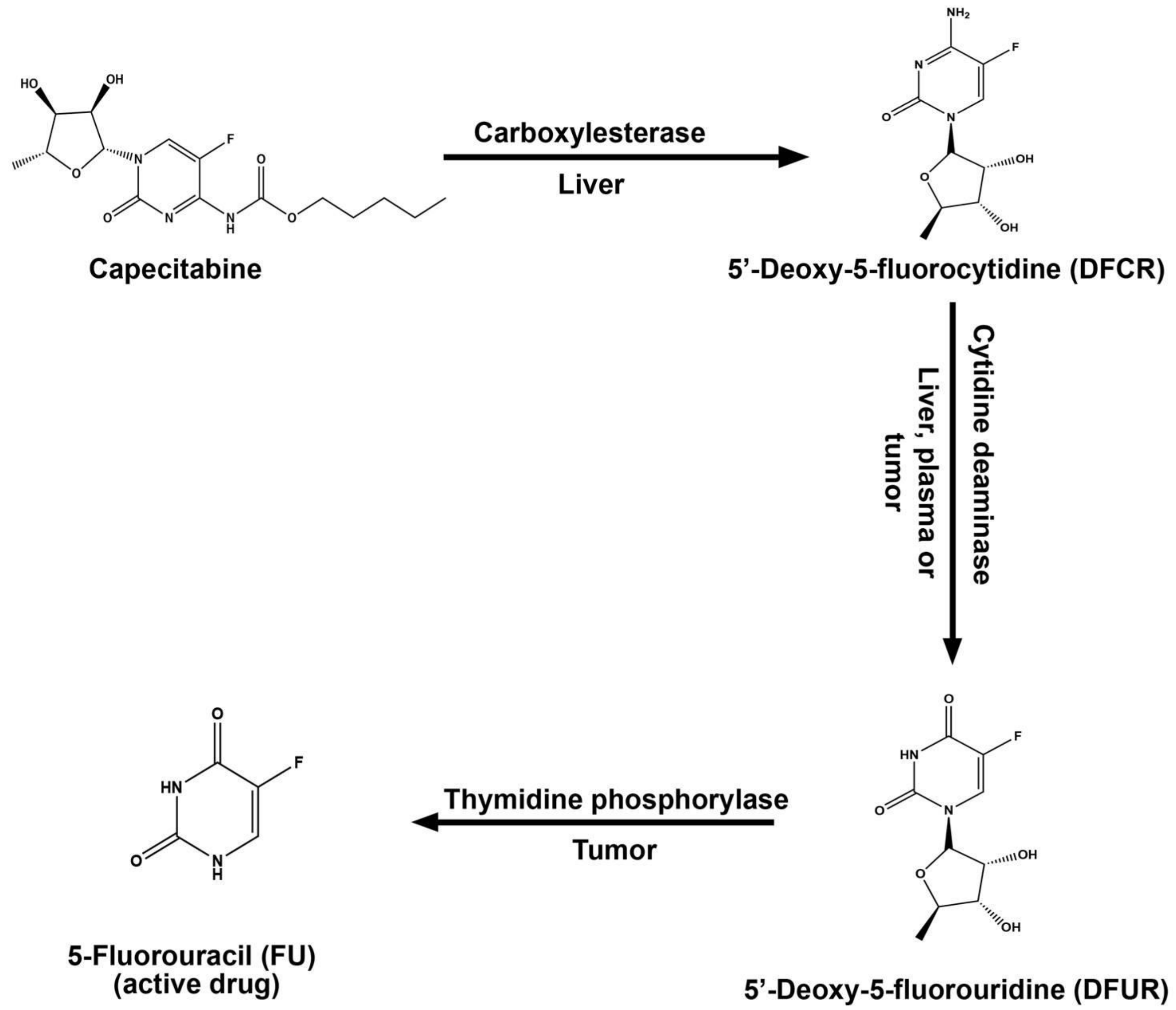

Scheme S1. Schematic of the action mechanism of the prodrug capecitabine.

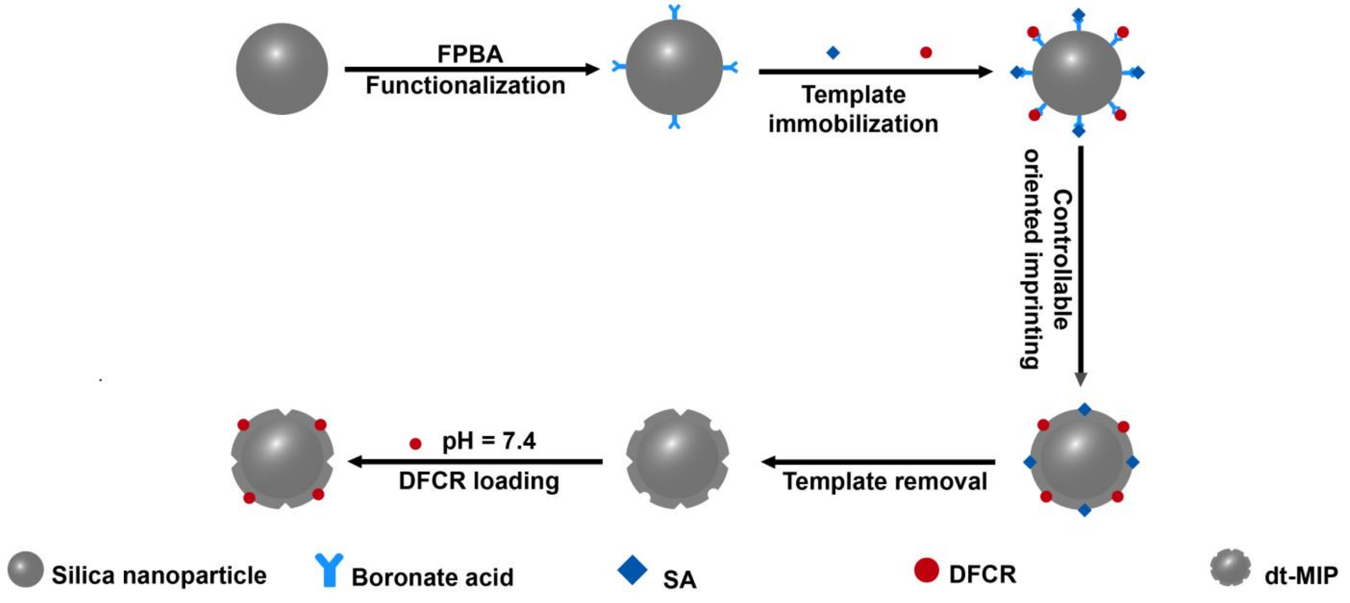

Scheme S2. Schematic of the synthesis route of dt-MIP and DFCR-loaded dt-MIP.

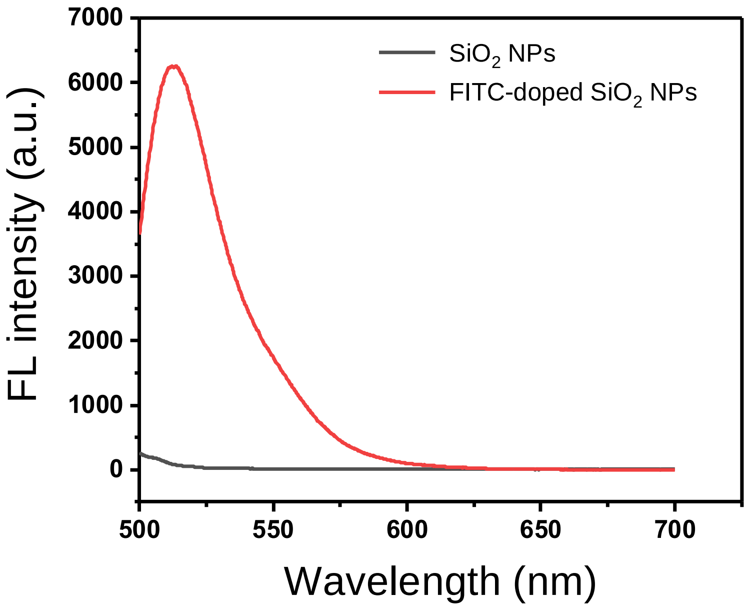

Figure S1. FL intensity of SiO_2 NPs and FITC-doped SiO_2 NPs. Excitation wavelength = 488 nm.

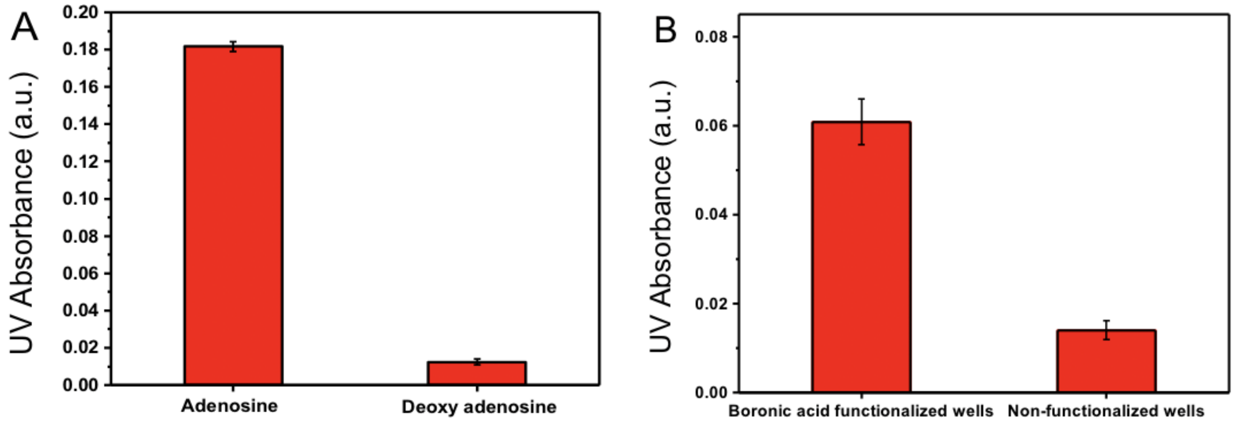

Figure S2. Characterization of the boronic acid-functionalization of A) nanoparticles, and B) 96-well.

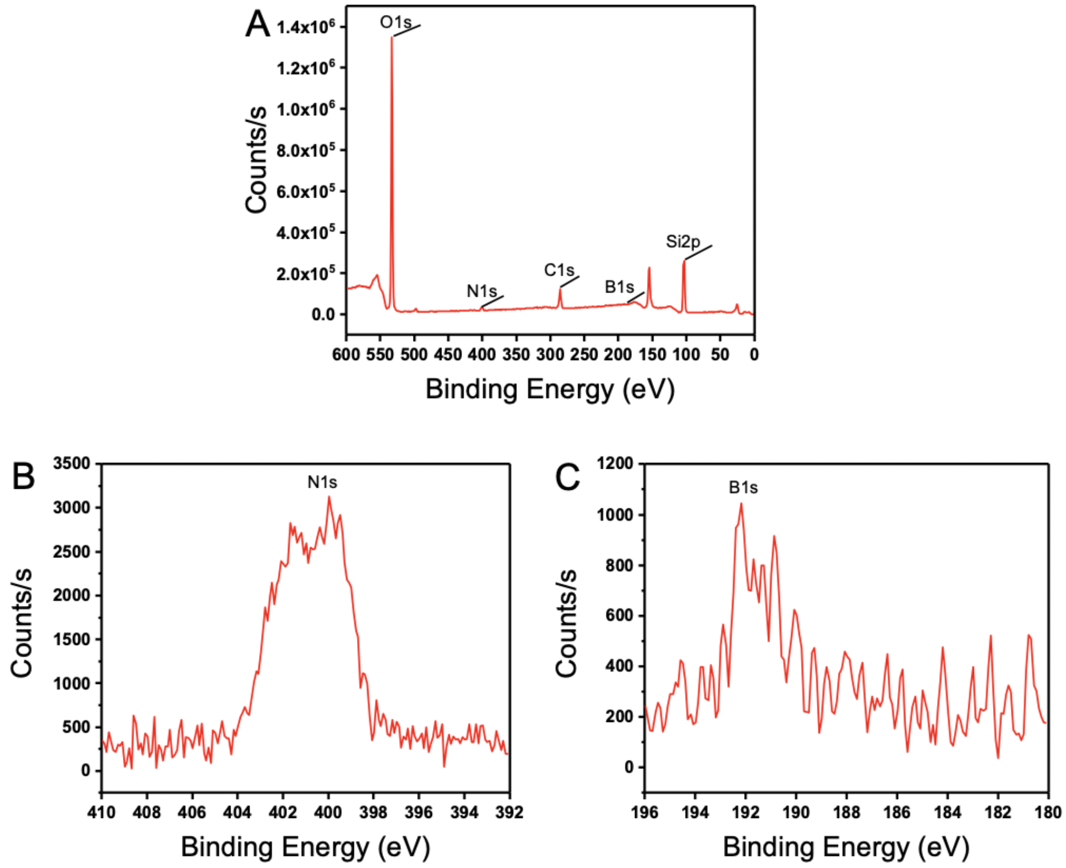

Figure S3. A) XPS survey spectrum of the FPBA-NPs. High-resolution XPS spectra of B) N1s and C) B1s.

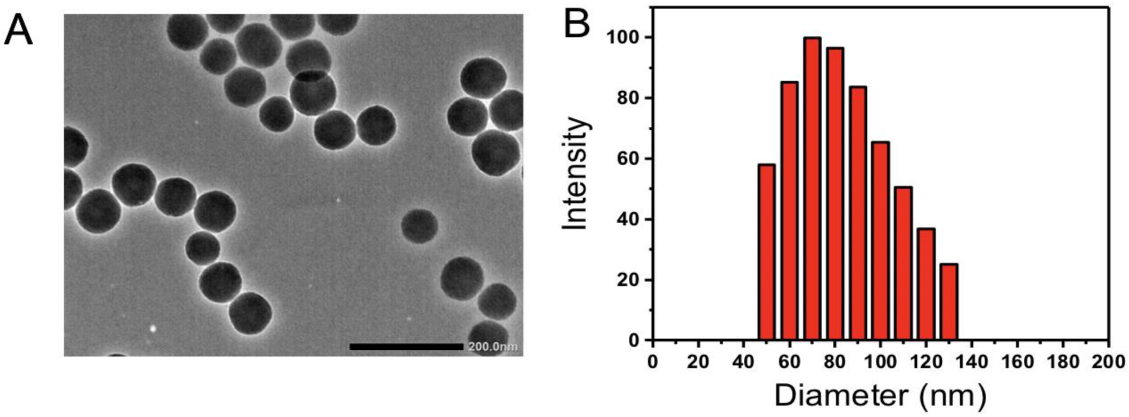

Figure S4. A) TEM image of dt-MIP; B) DLS characterization of dt-MIP.

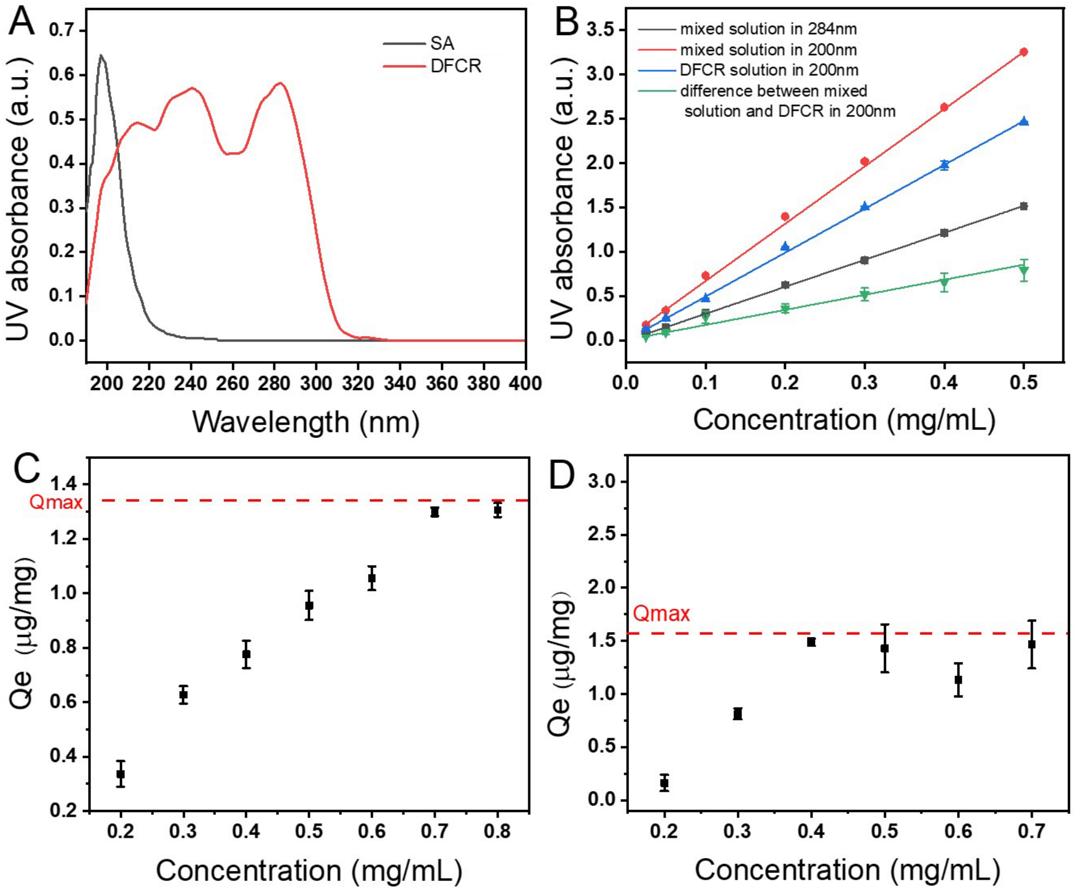

Figure S5. A) UV absorbance of DFCR and SA; B) Calibration curve of different solution; C) Adsorption isotherm of DFCR on FPBA-NPs determined in a mixed solution of DFCR and SA with equal concentration; D) Adsorption isotherms of SA on FPBA-NPs determined in a mixed solution of DFCR and SA with equal concentration. Qe: the amount of the target compound bound by FPBA-NPs at equilibrium.

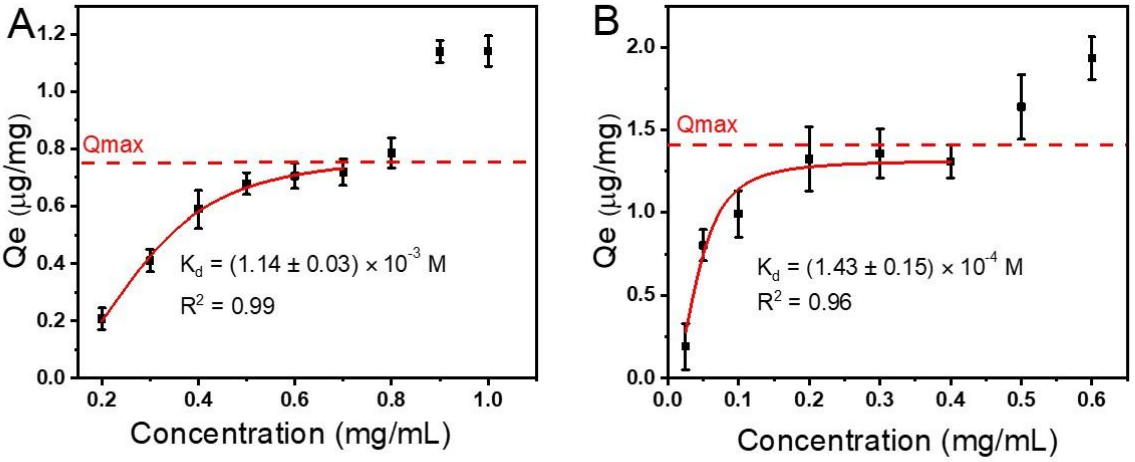

Figure S6. A) Adsorption isotherms of DFCR on dt-MIP; B) Adsorption isotherms of SA on dt-MIP. Red curve, data fitting by eq. (1). Qe: the amount of the target compound bound by dt-MIP at equilibrium.

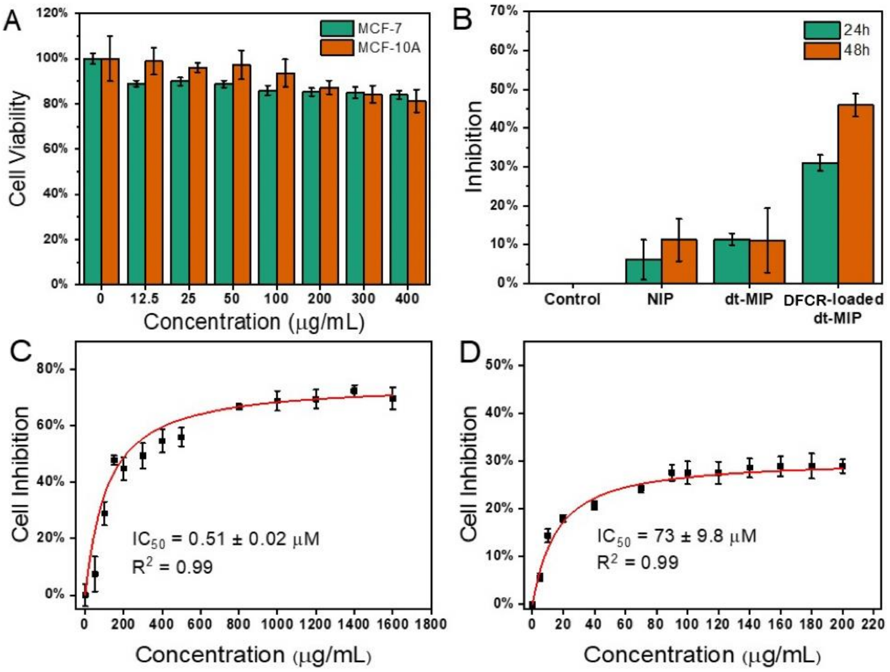

Figure S7. A) Cell viability of the breast cancer cell (MCF-7) and normal breast epithelial cell (MCF-10A) under treated by dt-MIP with different concentrations; B) Inhibition of MCF-7 cell growth by different materials of 200 μg /mL for 24 and 48 h; C) In vitro cytotoxicity data for DFCR-loaded dt-MIP in HepG-2 cell line; D) In vitro cytotoxicity data for DFCR in HepG-2 cell line. Red curve, data fitting by eq. (4).

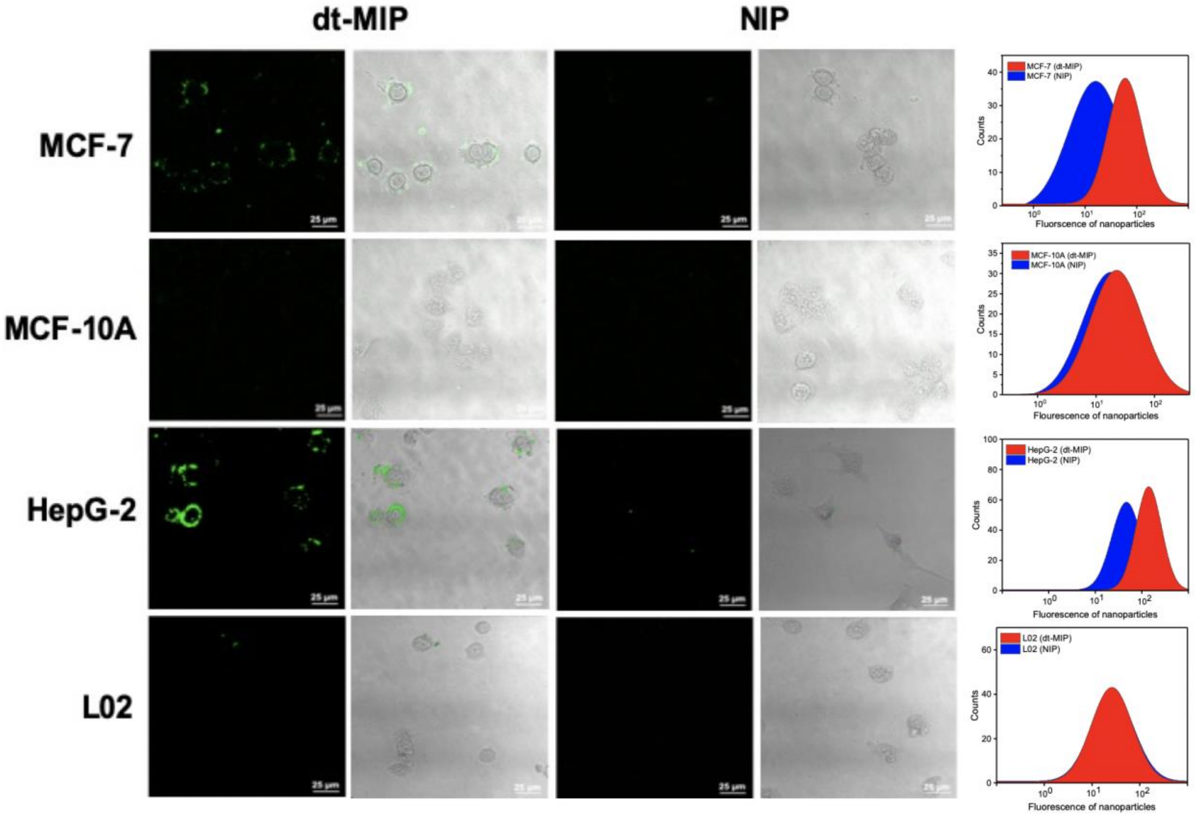

Figure S8. Confocal fluorescence imaging and flow cytometry of normal cell (MCF-10A and L02) and cancer cell (MCF-7 and HepG-2) after staining with dt-MIP and NIP. The concentration of the NPs was 200 μg/mL.

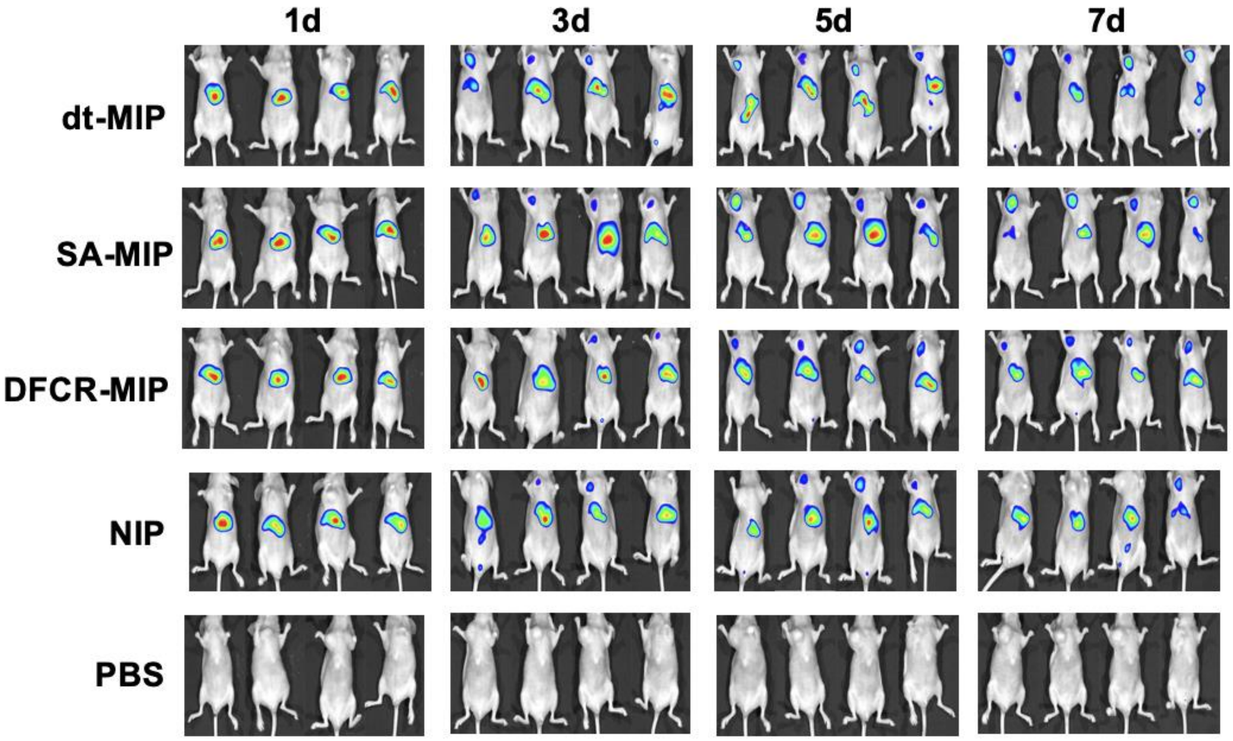

Figure S9. In vivo fluorescence imaging of HepG-2 tumor (left upper chest) and liver site (upper abdomen) after intravenous injection of NIR797-doped dt-MIP, SA-MIP, DFCR-MIP, NIP and PBS for different times.

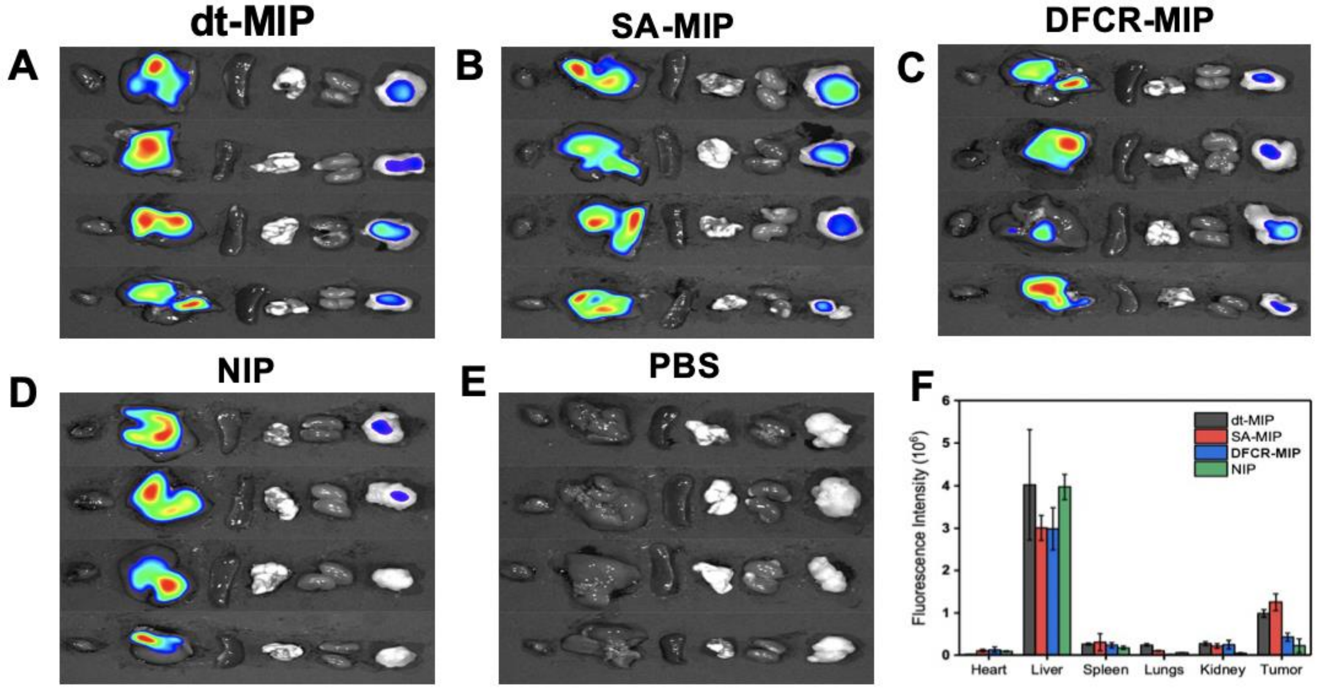

Figure S10. Fluorescence imaging of main organs (from left to right is heart, liver, spleen, lungs, kidney and tumor) surgically removed from the mice treated with different NPs for 7 days. mice treated with A) dt-MIP; B) SA-MIP; C) DFCR-MIP; D) NIP; E) PBS; F) the quantitative biodistribution of nanomaterials in the main organs (the fluorescence intensity was deducted using the PBS group as the reference).

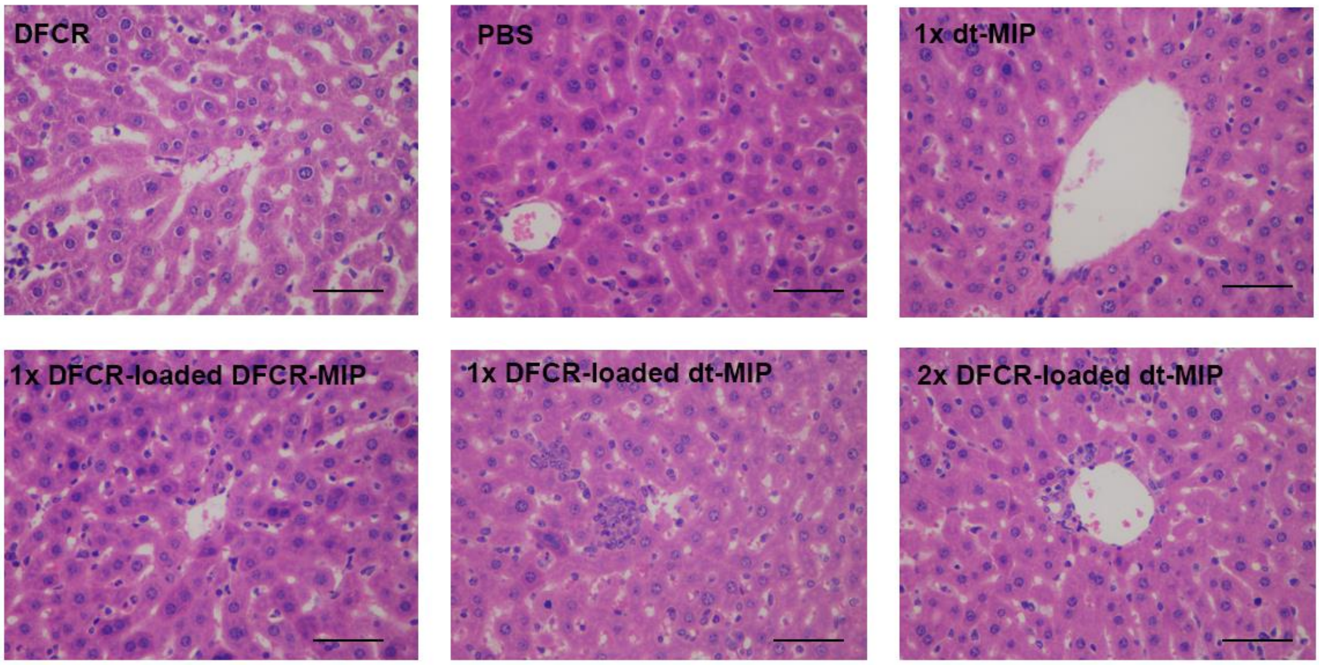

Figure S11. H&E staining of the liver of HepG-2 tumor-xenografted nude mice under different treatment. Scale bar = 50 μm.

Table S1. Summary of the contents of the Si, B, C, N, O elements in FPBA-NPs.

Surface Atomic Concentration (at. %)				
Si2p	B1s	C1s	N1s	O1s
28.3	1.09	11.06	1.39	58.16
